# Combined detection of inhibitors of the activin receptor signaling pathways (IASPs) by means of LC-HRMS/MS for human doping control

**DOI:** 10.1038/s41598-025-03562-y

**Published:** 2025-06-06

**Authors:** Panagiotis Sakellariou, Katja Walpurgis, Andreas Thomas, Alexandre Marchand, Geoff Miller, Frank Dellanna, Mario Thevis

**Affiliations:** 1 Institute of Biochemistry/Center for Preventive Doping Research, German Sport University Cologne, Am Sportpark Müngersdorf 6, 50933 Cologne, Germany; 2 Laboratoire Antidopage Français (LADF), Université Paris-Saclay, Orsay, France; 3 Sports Medicine Research and Testing Laboratory (SMRTL), South Jordan, Utah USA; 4 MVZ DaVita Rhein-Ruhr GmbH, Bismarckstraße 101, 40210 Düsseldorf, Germany; 5 European Monitoring Center for Emerging Doping Agents (EuMoCEDA), Cologne/Bonn, Germany

**Keywords:** Therapeutic antibodies, Doping, Activin receptor, Fusion protein, (Immuno-)affinity purification, LC-HRMS/MS, Transforming growth factor beta, Proteins, Liquid chromatography, Protein purification, Mass spectrometry

## Abstract

Members of the transforming growth factor beta superfamily such as myostatin, activin A, and GDF-11, are dimeric cytokines signaling through activin receptors. They play important regulative roles in different biological processes as the formation of muscle and red blood cells. Therefore, inhibitors of the activin receptor signaling pathways (IASPs) are potential performance-enhancing agents in sports, which are included in sections S2 (“Peptide hormones, growth factors, related substances and mimetics”) and S4 (“Hormone and metabolic modulators”) of the WADA Prohibited List. Within this research project, a multiplexed detection assay for nine IASPs in doping control serum/plasma samples by means of (immuno-)affinity purification, tryptic digestion and LC-HRMS/MS was developed. The method was validated and proved to be specific and sensitive (LOD: 10–50 ng/mL). Additionally, it was modified to allow for using urine. As proof-of-concept, authentic Luspatercept serum and urine and Sotatercept serum post-administration samples were successfully analyzed. Luspatercept could be detected in both matrices up to 70 days after the initial and 7 weeks after the second dose. Sotatercept was successfully detected in a serum sample collected 43 h following injection. The presented method can be employed in doping control routine analysis as a qualitative initial testing procedure.

## Introduction

The activin receptor signaling pathway plays an important role in the regulation of various biological processes, including muscle growth, tissue repair and differentiation of erythrocytes. The signaling through activin type II receptors (ActRIIA and ActRIIB) is activated by cytokines of the Transforming Growth Factor beta (TGF-β) superfamily, such as activin A (ActA), myostatin (MSTN) and Growth Differentiation Factor 11 (GDF-11)^1^. These ligands influence intracellular functions by binding to the extracellular domain of an activin type II receptor, enabling the type I receptor to also bind the ligand. When two pairs of type I and II receptors are combined, the homodimerization of the type II receptors promotes their kinase activity, resulting in the type I receptor’s transmembrane activin receptor-like kinase ALK4/5 activation, which in turn phosphorylates Smad2/3 transcription factors, and the Smad4 component is recruited. This Smad complex enters the nucleus and regulates downstream gene expression critical for cell growth, differentiation and apoptosis^1–3^.

Both MSTN and GDF-11 are negative regulators of muscle growth, and their binding to activin type II receptors suppresses myogenesis, limits muscle hypertrophy and promotes muscle wasting, which is often observed in various pathological conditions^4,5^. Additionally, receptor activation by ActA, MSTN and GDF-11 has been shown to impair red blood cell production, since late-stage erythropoiesis is erythropoietin-independent and TGF-β cytokines were found to negatively affect burst forming unit-erythroid (BFU-E) progenitors and late-stage precursor differentiation, resulting in diseases such as anaemia^1,6^. Therefore, inhibitors of the activin receptor signaling pathways (IASPs) have been tested as therapeutic strategies in muscle wasting diseases and disorders of erythropoiesis, but have also gained attention for their potential to promote muscle growth and improve athletic performance^3–5,7^. The use of IASPs as performance-enhancing agents presents both ethical and health concerns, so their use in sports is prohibited by the World Anti-Doping Agency (WADA) and they are included in sections S2 (“Peptide hormones, growth factors, related substances, and mimetics”) and S4 (“Hormone and metabolic modulators”) of the WADA Prohibited List^8^.

Inhibition of the activin receptor signaling pathways can be achieved through various approaches, with neutralization and interception of the receptor ligands emerging as promising therapeutic targets for the treatment of muscular diseases or diseases related to erythropoiesis^1,7^. As a result, pharmaceutical products based on these strategies have been tested in preclinical or clinical phases, while these products may also be of interest in sports doping. The neutralization of the TGF-β cytokines may occur by humanized or fully human therapeutic antibodies specifically targeting ActA, MSTN or GDF-11^4,7^. Garetosmab (REGN2477, Regeneron Pharmaceuticals), a monoclonal anti-ActA antibody, Stamulumab (MYO-029, Wyeth Pharmaceuticals), a recombinant human anti-MSTN antibody, Landogrozumab (LY2495655, Eli Lilly and Company), a humanized monoclonal anti-MSTN antibody and Domagrozumab (PF-06252616, Pfizer), a humanized monoclonal anti-MSTN antibody with high affinity to both MSTN and GDF-11, are the most extensively researched candidate drugs for neutralizing TGF-β cytokines^1,4,5,7,9–12^. Additionally, both ActA and MSTN exist in precursor forms before being cleaved into their mature, active states. Inhibition can be targeted during the processing of these precursors, preventing their maturation into fully active proteins^2,13^. Apitegromab (SRK-015, Scholar Rock) is a monoclonal antibody that uniquely blocks MSTN activation by capturing both its pro- and latent forms, preventing the cleavage of latent MSTN into active MSTN, without any affinity for mature MSTN, ActA or GDF-11^1,2,13,14^.

Interception of the receptor ligands can be adopted by fusion proteins from the extracellular domain of activin receptors and a human immunoglobin Fc fragment, which have been developed to act as decoy receptors or ligand traps^7^. Sotatercept (ACE-011, Acceleron Pharma), a glycosylated dimeric Fc fusion protein, consists of the extracellular domain of ActRIIA fused to the Fc fragment of human immunoglobin G1 (IgG1) and has a ligand binding moiety identical to the native receptor, with a single-amino-terminal truncation. This protein drug acts as a decoy receptor with higher affinity to ActA and GDF-11 than to MSTN, affects late-stage erythropoiesis, enhancing red blood cell production, and increases bone formation^1,7,15^. Similarly, Elritercept (KER-050, Keros Therapeutics), a recombinant fusion protein comprising a modified ActRIIA extracellular domain fused to human IgG1 Fc, acts as a soluble ligand trap to inhibit mostly ActA^16^. Luspatercept (ACE-536, Acceleron Pharma) is a modified ActRIIB-Fc molecule with amino- and carboxy-terminal truncations. In contrast to the endogenous receptor, an amino acid substitution (L79D) was introduced to reduce its affinity for ActA and bone morphogenetic proteins (BMPs), resulting in preferential binding to GDF-11 and MSTN. This drug acts, similar to Sotatercept, as a decoy receptor promoting erythropoiesis^1,6,7,17^. Ramatercept (ACE-031, Acceleron Pharma) is a soluble ActRIIB-Fc ligand trap identical to Luspatercept but without the L79D substitution, which blocks the interaction of ActA, MSTN and GDF-11 with the endogenous receptor^5,18^.

Over the past few years, doping control detection methods for the monitoring of IASPs misuse in human sports have been developed. This is especially important, because reference materials for these emerging drugs are frequently available for research purposes or may be traded on the black market, whether or not the drug has received clinical approval^7^. The majority of the described methods refer to the detection of Sotatercept and/or Luspatercept in serum or plasma samples using affinity or immunoaffinity purification followed by liquid chromatography–high resolution (tandem) mass spectrometry (LC-HR(MS)/MS) or electrophoretic techniques and Western blotting^19–24^. Furthermore, a commercial anti-ActRIIA ELISA was evaluated for use in sports drug testing and found to be suitable for detecting Luspatercept in serum^25^. Additionally, Sotatercept and Luspatercept detection methods for different matrices such as Dried Blood Spots (DBS) and urine have been described^26,27^. For the other IASPs, only approaches targeting Stamulumab or Domagrozumab in serum or plasma using Western blotting and LC-HRMS are known^28,29^. Recently, a method for the detection of anti-MSTN antibodies, including Landogrozumab and Domagrozumab, in DBS and plasma using LC–MS/MS was described^30^.

**Fig. 1:**
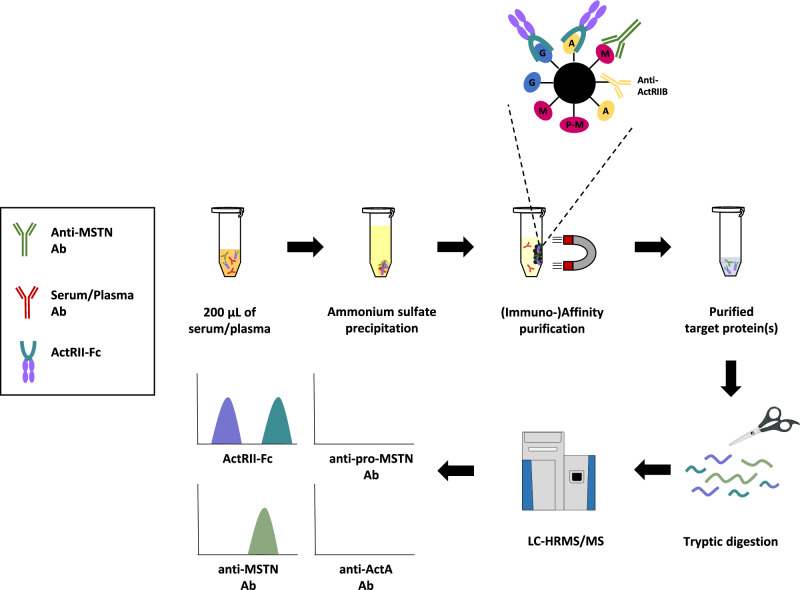
Method overview. A: activin A (ActA), G: Growth Differentiation Factor 11 (GDF-11), M: myostatin (MSTN), P-M: pro-MSTN, ActRIIB: activin type II receptor B, Ab: antibody, Fc: immunoglobin Fc fragment, LC-HRMS/MS: liquid chromatography–high resolution tandem mass spectrometry.

The aim of this research project was the development of a multiplexed assay for the combined detection of the nine different IASPs mentioned above in doping control serum or plasma samples using (immuno-)affinity purification, tryptic digestion and LC-HRMS/MS (Fig. 1). Following assay optimization, the method was validated according to WADA guidelines^31^. Post-administration human serum samples collected after Luspatercept and Sotatercept injection(s) were analysed as proof-of-concept. The method was modified to also be applicable to urine sample analysis, and Luspatercept post-administration urine samples were successfully extracted.

## Material & methods

### Reference material, chemicals & consumables

Garetosmab, Landogrozumab and Stamulumab reference materials were purchased from MedChemExpress (New Jersey, USA). Sotatercept, Elritercept, Luspatercept, Ramatercept and Apitegromab reference materials were purchased from ProteoGenix (Strasbourg, France). A solution containing 10 mg/mL of Domagrozumab was kindly provided by Pfizer (New York, USA). A recombinant fusion protein composed of the extracellular domain of mouse ActRIIA and the Fc region of mouse IgG2A (mActRIIA-Fc) was used as internal standard (ISTD) and obtained from R&D Systems (Minnesota, USA)^19^. Magnetic beads for affinity purification were prepared by using NHS Magnetic Sepharose from Cytiva (Massachusetts, USA), ActA, GDF-11, MSTN from Peprotech (Munich, Germany), latent MSTN from Kactus (Paris, France) and a goat anti-human ActRIIB antibody (AF339) from R&D Systems (Minnesota, USA). Reagents required for protein reduction and alkylation were tris(2-carboxyethyl)phosphine (TCEP) and iodoacetamide (IAA), and were purchased from Merck (Darmstadt, Germany) and Thermo Fisher Scientific (Massachusetts, USA), respectively. Sequencing grade modified trypsin (V5111) was obtained from Promega (Wisconsin, USA). Protein low bind tubes were bought from Eppendorf (Hamburg, Germany) and the magnetic rack from Thermo Fisher Scientific (Massachusetts, USA). Amicon^®^ Ultra-4 centrifugal filter units (cut-off: 10 kDa) were from Merck (Darmstadt, Germany), as all other chemicals and solvents that were of analytical grade.

### Samples

#### Samples used for method development and validation

For the development and characterization of the LC-HRMS/MS assay, commercially available human serum obtained from male AB plasma was purchased from Merck (Darmstadt, Germany). For method validation, blood samples were additionally collected from 10 healthy volunteers (5 males and 5 females) in EDTA and serum separator gel tubes. EDTA tubes were centrifuged for 20 min at 1300 × *g* to isolate plasma, while serum separator gel tubes were centrifuged for 20 min at 1300 × *g*, after clotting for 30 min at room temperature (RT) to separate serum. For urine method optimization, a urine sample was collected from one healthy male volunteer. The blank sample collection and use were approved by the local ethics committee of the German Sport University Cologne (139/2021), whose guidelines and regulations were applied to the entire research project and all included methods, and all participants provided written informed consent.

#### Luspatercept administration study

Aliquoted samples from a Luspatercept administration study were kindly provided by the Paris Anti-Doping Laboratory, where they had been already analyzed by electrophoretic techniques^27^. As described by Marchand et al., four healthy volunteers (2 males and 2 females, aged 18–50 years) received Reblozyl^®^ (Luspatercept, Acceleron Pharma) by subcutaneous injection in the abdomen at a dose of 0.25 mg/kg body weight. This dose was the highest well-tolerated dose during the Phase I clinical study. The study was conducted by the Sports Medicine Research and Testing Laboratory (South Jordan, Utah, USA) and included two administrations, separated by 3 weeks, according to the recommended therapeutic treatment. All subjects provided written informed consent. Aliquoted serum samples collected 4 days pre-administration and 2, 21, 28, 63 and 70 days post-administration (referring to the first dose), as well as aliquoted urine samples collected 4 and 2 days pre-administration and 2, 21, 28, 63 and 70 days post-administration (referring to the first dose) from four volunteers (M3, M4, F1 and F6) were sent to the Cologne Anti-Doping Laboratory (except for subject F6; urine sample 35 days post-administration instead of 28 days), and stored at − 20 °C until analysis.

#### Sotatercept administration study

A female patient received a single subcutaneous injection of Sotatercept (Acceleron Pharma) at a dose of 0.5 mg/kg body weight for therapeutic reasons. A serum sample was collected 43 h post-administration, sent to the Cologne Anti-Doping Laboratory, and stored at − 20 °C until analysis. The volunteer provided written informed consent for using the sample for method development and research purposes.

### Detection of different IASPs in serum or plasma by means of (immuno-)affinity purification, tryptic digestion and LC-HRMS/MS

#### Magnetic beads preparation

Magnetic beads conjugated with human ActA, GDF-11, MSTN, pro/latent MSTN and a goat anti-human ActRIIB antibody were utilized for (immuno-)affinity purification. Beads preparation was carried out following the manufacturer’s instructions and as outlined in a previous study^19^. Ten batches of immuno-purification resin were prepared in parallel by transferring 25 μL of NHS Magnetic Sepharose slurry to a 1.5 mL protein Lo-Bind tube and, with the application of a magnetic rack, the storage solution was removed. Following equilibration with 500 µL of ice-cold 1 mM HCl, magnetic beads were incubated for 30 min at 1200 rpm and RT with 2 µg each of ActA, GDF-11 and MSTN, diluted in 20 µL of Coupling Buffer (0.2 M NaHCO_3_, 0.5 M NaCl, pH 8.3) and 10 µg each of latent MSTN and goat anti-human ActRIIB antibody, diluted in 30 µL of Coupling Buffer. The supernatant was removed using the magnetic rack and beads were sequentially incubated with 500 μL of Blocking Buffer A (50 mM Tris–HCl, 1 M NaCl, pH 8.0) and Blocking Buffer B (50 mM glycine, 1 M NaCl, pH 3.0), three times per buffer. The second washing step with Blocking Buffer A was conducted using a rotating sample mixer for 15 min at RT. After removing the supernatant, the beads were then equilibrated twice in 500 μL of PBST (phosphate-buffered saline, PBS; 1 tablet per 200 mL of deionized water: 0.01 M phosphate buffer, 0.0027 M potassium chloride and 0.137 M sodium chloride, pH 7.4, with 0.1% Tween 20) and reconstituted with 300 μL of PBST. Ultimately, the conjugated magnetic beads were pooled in a 5 mL protein Lo-Bind tube and stored at 4 °C until usage.

#### Sample extraction

For sample extraction, a protocol described by Walpurgis et al. in 2016 was used and slightly modified regarding washing conditions^19^. Prior to (immuno-)affinity purification, 200 μL of human serum or plasma, fortified with 40 ng of the internal standard (ISTD: 4 μL of a solution containing 10 μg/mL of mActRIIA-Fc) were diluted with 600 μL of PBS and saturated ammonium sulfate solution was added in a 1:1 ratio to precipitate any antibodies and IgG-based fusion proteins contained. After incubation for 30 min at RT, samples were centrifuged for 10 min at 10,000 × *g* and the remaining serum or plasma components were removed. Following pellet reconstitution with 200 μL of PBST, samples were mixed with 300 μL of the conjugated magnetic beads and (immuno-)affinity purification was conducted using a rotating sample mixer for at least 60 min at RT. Subsequent to the incubation, samples were washed once with 500 µL of PBST and twice with 500 µL of PBS, in order to remove proteins non-specifically bound to the surface of the beads. The elution of the ligands specifically bound by the beads was performed with 50 µL of 3% acetic acid for 15 min at 1200 rpm and RT, as the receptor-ligand interaction is disrupted by lowering the pH value to 2.5. Then, magnetic beads were washed three times with 300 µL of 3% acetic acid and three times with 500 µL of PBST to allow for their re-usage. Finally, they were resuspended in 300 µL of PBST and pooled with the magnetic beads stock solution stored at 4 °C.

#### Tryptic digestion

For specific and sensitive mass spectrometric detection, the protein-based target analytes were proteolytically cleaved into smaller peptides. Here, a protocol successfully used in earlier research projects was employed and further modified regarding the amounts of neutralization buffer, IAA, ACN & trypsin^19,22,29,32^. The reduction of the disulfide bonds in the proteins of the sample extracts obtained from (immuno-)affinity purification, was conducted with 500 nmol of TCEP (5 µL of a 0.1 M solution) for 15 min at 900 rpm at 60°C^32^. The reduced acidic eluates were neutralized with 25 µL of 2 M NH_4_HCO_3_ to adjust the pH value for tryptic digestion. The alkylation of the reduced disulfide bridges was performed with 1,875 nmol of IAA (7.5 µL of a 0.25 M solution in 100 mM NH_4_HCO_3_) for 30 min at RT in the dark. For tryptic digestion, 2 µg of trypsin (50 µL of a 40 µg/mL solution in 50 mM NH_4_HCO_3_ with 0.01% glacial acetic acid) and 15 µL of acetonitrile (ACN) were added, the latter to increase efficiency through protein denaturation, and samples were incubated overnight at 37 °C and 500 rpm. To stop tryptic digestion, samples were mixed with 5 µL of glacial acetic acid, transferred to polypropylene high-performance liquid chromatography (HPLC) vials and subjected to LC-HRMS/MS analysis.

#### LC-HRMS/MS

LC-HRMS/MS analysis was performed using a Vanquish UHPLC system coupled to an Orbitrap Exploris™ 480 mass spectrometer (Thermo Fisher Scientific). The chromatographic separation was carried out with a dual-pump set-up and trapping of the analytes was conducted on an Accucore™ Phenyl-Hexyl pre-column (3 × 10 mm, 2.6 μm) (Thermo Fisher Scientific). The solvents employed for trapping were 0.1% formic acid in water (A1) and 0.1% formic acid in ACN (B1). After trapping for 2 min at 99% of solvent A1, the flow was switched to a Poroshell EC-C18 analytical column (3 × 50 mm, 2.7 μm) (Agilent Technologies, California, USA). The column temperature was set to 30 °C, the autosampler temperature to 10 °C, and the injection volume was 10 μL. The mobile phase consisted of 0.1% formic acid in water with 1% dimethyl sulfoxide (DMSO) as solvent A, and 0.1% formic acid in ACN with 2% DMSO as solvent B. The LC gradient was set with a flow of 0.45 mL/min and run with a total run time of 17 min as follows: 0–2 min isocratic trapping with 1% B1, 2–3 min 1–15% B, 3–7 min 15–25% B, 7–11 min 25–35% B, 11–13 min 35–80% B, 13–17 min re-equilibration with 1% B.

The mass spectrometer was operated in positive ionization mode with an ionization voltage of 4000 V and an ion transfer tube temperature of 320 °C. The full scan experiments were conducted with a range of *m/z* 400–2400 at a resolution of 60,000 full width at half maximum (FWHM) at *m/z* 200. Targeted selected ion monitoring (tSIM) experiments with an isolation window of *m/z* = 3 and a resolution of 45,000 FWHM at *m/z* 200 were conducted using an inclusion list with the accurate mass-to-charge ratios for the most abundant charge states of the target peptides (Table 1). In addition, data-dependent MS/MS (ddMS^2^) scans with an isolation window of *m/z* = 2, a first mass of *m/z* = 200, and an intensity threshold of 5.0E3 were recorded at a resolution of 15,000 FWHM at *m/z* 200 using a higher-energy collisional dissociation (HCD) collision energy (CE) of 30% and nitrogen obtained from an N_2_-generator (CMC, Eschborn, Germany) as collision gas. The MS was calibrated according to the manufacturer’s recommendations with a mixture of caffeine, the tetrapeptide MRFA (methionine-arginine-phenylalanine-alanine), and Ultramark 1621 (Thermo Fisher Scientific). The acquired MS data were evaluated using Thermo Xcalibur Software (Version 4.0.27.10, 2015, Thermo Fisher Scientific).

**Table 1:** Target peptides included in the LC-HRMS/MS ITP for IASPs.

Drug candidate	Drug class	Ligand(s)	Subunit	Peptide#	AA#	Amino acid sequence	Monoisotopic mass [*m/z*]	Target Ion [*m/z*]	Charge	RT [min]
Garetosmab	Antibody	ActA	HC	T_3_	44–64	GLEWIGYILYTGGTSFNPSLK	1158.60	1159.1029	2	11.5
			LC	T_7_	79–104	LEPEDFAVYYCQQYGSSPWTFGQGTK	1529.68	1530.1857	2	9.0
Sotatercept	Fusion protein	ActA, GDF-11	ActRIIA	T_2_	5–19	SETQECLFFNANWEK	951.92	952.4205	2	7.5
			Fc	T_24_	224–231	ALPVPIEK	433.77	433.7723	2	4.8
Elritercept	Fusion protein	ActA, MSTN, GDF-11	modActRIIA	T_2_	7–23	SETQECLFYNANWELER	1094.98	1095.4860	2	7.5
			modActRIIA	T_7_	40–47	LHCYATWR	553.76	553.7641	2	4.0
			modActRIIA/ActRIIB	T_10_	59–70	GCWLDDFNCYDR	810.81	810.8147	2	7.2
			modActRIIA	T_11_	71–95	TDCVETEENPQVYFCCCEGNMCNEK	1587.09	1588.0937	2	5.7
Luspatercept	Fusion protein	GDF-11, MSTN	ActRIIB	T_2_	4–16	ECIYYNANWELER	880.39	880.8912	2	6.2
			ActRIIB	T_6_	33–40	LHCYASWR	546.76	546.7565	2	3.9
			modActRIIB	T_9_	52–63	GCWDDDFNCYDR	811.79	811.7860	2	5.5
			ActRIIB	T_10_	64–88	QECVATEENPQVYFCCCEGNFCNER	1600.62	1601.6214	2	6.2
Ramatercept	Fusion protein	MSTN, GDF-11, ActA	ActRIIB	T_2_	4–16	ECIYYNANWELER	880.39	880.8912	2	6.2
			ActRIIB	T_6_	33–40	LHCYASWR	546.76	546.7565	2	3.9
			modActRIIA/ActRIIB	T_9_	52–63	GCWLDDFNCYDR	810.81	810.8147	2	7.3
			ActRIIB	T_10_	64–88	QECVATEENPQVYFCCCEGNFCNER	1600.62	1601.6214	2	6.2
Apitegromab	Antibody	Latent/Pro-MSTN	LC	T_2_	18–46	VTISCSGSSSNIGSNTVHWYQQLPGTAPK	1026.17	1026.4979	3	6.3
			LC	T_3_	47–62	LLIYSDNQRPSGVPDR	915.48	915.4771	2	4.5
Landogrozumab	Antibody	MSTN	HC	T_5_	44–65	GLVWVSAITSSGGSTYYSDTVK	1139.57	1140.0689	2	8.2
			LC	T_4_	47–62	LLIYSTSNLVAGIPDR	866.49	866.4865	2	7.8
Stamulumab	Antibody	MSTN	LC	T_1_	1–30	SYELTQPPSVSVSPGQTASITCSGHALGDK	1025.50	1025.8259	3	5.7
			LC	T_2_	31–60	FVSWYQQKPGQSPVLVIYDDTQRPSGIPER	1164.26	1164.6054	3	7.6
Domagrozumab	Antibody	MSTN, GDF-11	HC	T_4_	44–65	GLEWVSTISSGGSYTSYPDSVK	1160.55	1161.0564	2	7.4
			HC	T_18_	222–247	THTCPPCPAPEAAGAPSVFLFPPKPK	925.46	925.8005	3	6.4
Mouse ActRIIA-Fc	Fusion protein	ActA	mouse ActRIIA	T_3_	25–39	SETQECLFFNANWER	965.92	966.4237	2	7.6
			mouse Fc	T_33_	320–336	NTEPVLDSDGSYFMYSK	976.93	977.4350	2	6.5

*HC* heavy chain, *LC* light chain, *Fc* immunoglobin Fc fragment, *ActRIIA/B* activin type II receptor A/B, *mod* modified, *AA* amino acid, *RT* retention time.

#### Method validation

Method validation was conducted according to current WADA guidelines and the analytical parameters were evaluated for all target peptides shown in Table 1^31^. The samples for the method validation, unless anything else is mentioned, were prepared with the already described preparation procedure. The selectivity of the method was demonstrated by analyzing 10 different blank serum samples (5 male, 5 female) fortified only with the ISTD. To prove the assay’s reliability, these 10 different serum samples were spiked with the nine target IASPs at a concentration of 100 ng/mL each and analyzed, as described above. Likewise, 6 of these serum samples (3 males, 3 females) were analyzed to determine the limit of detection (LOD), after being fortified with the nine IASPs at four different concentrations (10, 25, 50 and 100 ng/mL). Reliability sample extracts were stored in the autosampler of the LC–MS system at a temperature of 10 °C for a total of 3 days and subsequently re-analyzed to determine analyte stability. Furthermore, the risk for sample carryover during LC-HRMS/MS analysis was assessed by injecting a blank serum extract immediately after the extract of a serum sample fortified with the nine target IASPs at a concentration of 500 ng/mL. The same sample was used to investigate the sample carryover due to the magnetic beads’ re-use. In more detail, after the magnetic beads’ elution, they were washed three times with 300 µL of 3% acetic acid, and for each wash fraction, 50 µL were included in the preparation procedure, undergoing protein reduction, neutralization, alkylation, tryptic digestion, and LC-HRMS/MS analysis. To evaluate the robustness of the method, 10 different plasma samples (5 male, 5 female) were spiked with the nine target IASPs at a concentration of 100 ng/mL each and analyzed, as described above. Additionally, linearity was assessed by analyzing 8 serum specimens fortified with the target IASPs at a concentration of 0, 10, 25, 50, 75, 100, 250 and 500 ng/mL. Absolute peak areas were used to construct a calibration curve and determine linearity by regression analysis. Linearity sample extracts were stored in the autosampler of the LC–MS system at a temperature of 10 °C for a total of 3 days and subsequently re-analyzed to assess analyte stability at low concentrations.

#### Analysis of Luspatercept and Sotatercept administration study samples

As proof-of-concept for the method described, Luspatercept pre- and post-administration serum samples were analyzed. To avoid the risk of carryover due to the highly concentrated serum samples, they were diluted with commercially available human blank serum with the following ratios; 1:2 for samples collected 2, 63, and 70 days post-administration, and 1:4 for those collected 21 and 28 days post-administration. Additionally, one Sotatercept post-administration sample was analyzed. A single-point calibrator fortified with Luspatercept or Sotatercept at a concentration of 100 ng/mL was prepared with each sample batch.

### Detection of Luspatercept in urine by means of ultrafiltration, (immuno-)affinity purification, tryptic digestion and LC-HRMS/MS

The previously described method was modified to also be applicable for urine sample analysis, instead of serum^33^. During method development and optimization, 2 mL of blank urine samples spiked with the nine target IASPs at three different concentrations (50, 100 and 250 ng/mL) were tested. The samples were also spiked with 400 ng of the ISTD (4 μL of a solution containing 10 μg/mL of mouse Sotatercept). Instead of the ammonium sulfate precipitation, the urine samples were ultrafiltrated, using Amicon^®^ Ultra-4 centrifugal filter units with a cut-off of 10 kDa for 10 min at 4000 × *g*, and the retentates were washed with 4 mL of PBST for 20 min at 4000 × *g*. The washed retentates were subjected to (immuno-)affinity purification and all subsequent steps of the established procedure. The only other modification, compared to the serum sample preparation procedure, was the more intense washing of the beads, six times with 500 µL of 3% acetic acid and three times with 500 µL of PBST, to allow for their re-usage. Following method optimization, the Luspatercept pre- and post-administration urine samples were analysed and a single-point calibrator fortified with Luspatercept at a concentration of 10 ng/mL was prepared with each sample batch.

## Results & discussion

### Combined extraction of different IASPs from biological matrices

To allow for a multiplexed detection of different IASPs from doping control samples, a combined extraction from serum or plasma was planned by employing their high affinity for different ligands and coupling them to NHS Magnetic Sepharose beads. As described, the therapeutic antibodies Garetosmab and Apitegromab specifically bind to dimeric ActA and pro- and latent MSTN, respectively, while Landogrozumab, Stamulumab and Domagrozumab are anti-MSTN antibodies, with the last one also exhibiting high affinity for GDF-11. By contrast, the fusion proteins Sotatercept, Elritercept, Ramatercept and Luspatercept simultaneously target multiple TGF-β cytokines including ActA, MSTN, and GDF-11 (Table 1). Unfortunately, initial tests with the different target analytes and magnetic beads coupled separately to the different antigens resulted in insufficient recoveries of Luspatercept. Reference materials purchased from different manufacturers (Reblozyl^®^, Bristol-Myers Squibb; Luspatercept Biosimilar, ProteoGenix; Luspatercept, MedChemExpress) were tested and similar results were obtained, indicating that quality issues with the reference material are rather unlikely. According to early literature on Luspatercept, the modified ActRIIB-Fc fusion protein should bind strongly to the TGF-β cytokine GDF-11^34^. However, a more recent study also observed that the used Luspatercept reference material from Creative Biomart (New York, USA) exhibited no visible affinity for GDF-11 and other TGF-β cytokines^22^. In 2019, Guerra et al. could show that a lack of GDF-11 in transgenic mice does not reduce the effectiveness of the murine Luspatercept analog RAP-536, also suggesting that the main ligand of the fusion protein could be a different cytokine^35^. To facilitate an efficient extraction of Luspatercept from serum, the goat anti-human ActRIIB antibody successfully employed in earlier research projects was eventually selected^21–23^. The simultaneous use of different TGF-β cytokine ligands for affinity purification allows not only for an efficient extraction of the IASPs listed in Table 1, especially of those showing affinities toward multiple TGF-β cytokines, but also similar drugs that specifically bind to one of the cytokines. As the LC-HRMS/MS method used for analyte detection also acquires full MS data, a retrospective evaluation to investigate the prevalence of novel IASPs co-extracted by the multi-ligand magnetic beads would be possible.

### Identification of the target peptides for the unambiguous mass spectrometric detection of different IASPs

An unambiguous mass spectrometric identification of the nine therapeutic (candidate) drugs required unique diagnostic target peptides, which are ideally not present in any naturally occurring (human) protein or other protein drug. For this purpose, the amino acid sequences of the different target proteins were retrieved from various online databases and subjected to in silico tryptic digestion using GPMAW software (Version 8.00, Lighthouse data, Odense, Denmark). With the resulting tryptic peptides, ProteinBlast database searches against UniProtKB/Swiss-Prot and ‘nr’ were conducted to probe for their natural occurrence in human serum or plasma. The UniProtKB/Swiss-Prot database currently comprises more than 484,000 entries and is known to be highly curated and highly cross-referenced^36,37^. By contrast, the National Center for Biotechnology Information (NCBI) default protein database ‘nr’ is significantly larger, with more than 861,000,000 entries derived from different sources such as SwissProt, PIR and PDB databases^36,37^. Subsequently, in-solution tryptic digests of the different target proteins were analyzed by LC-HRMS/MS to verify the detectability of the resulting diagnostic peptides. The amino acid sequences and monoisotopic masses of the peptides eventually selected for protein detection are summarized in Table 1 and the corresponding MS/MS spectra shown in Supporting Information Figs. S1–20.

Especially for humanized or fully human therapeutic antibodies, the identification of proteotypic peptides can be challenging as they commonly differ only in the antigen-binding variable domains of both the heavy and light chains from the plethora of endogenous IgGs. For Garetosmab, a fully human antibody, the ProteinBlast search yielded only three tryptic peptides without matches in the UniProtKB/Swiss-Prot and ‘nr’ databases: Peptides HC-T_3_ GLEWIGYILYTGGTSFNPSLK, HC-T_5_ VSMSVGTSK and HC-T_9_ SGITFTGIIVPGSFDIWGQGTMVTV SSASTK. While peptide HC-T_3_ was sensitively detected in in-solution digests of Garetosmab reference material, peptides HC-T_5_ and HC-T_9_ were unfortunately characterized by significantly lower abundance, which can potentially be attributed to the presence of methionine residues susceptible towards oxidation during sample preparation (e.g. tryptic digestion or electrospray ionization (ESI)) or the presence of missed cleavage sites in the generated tryptic peptides. Therefore, an alternative second target peptide was required. For peptide LC-T_7_ LEPEDFAVYYCQQYGSSPWTFGQGTK, the ‘nr’ database search showed that this peptide could theoretically be present in the immunoglobulin kappa light chain variable region of human individuals. However, no hit was obtained using the UniProtKB/Swiss-Prot database, and also the analysis of an exemplary blank serum extract suggested the specificity of this peptide. Therefore, peptides HC-T_3_ and LC-T_7_ were finally included into the method and further evaluated during method validation. For confirmation purposes, also peptides HC-T_5_ and HC-T_9_ could be considered. The ProteinBlast of the tryptic peptides derived from the HC and LC of the humanized antibody Landogrozumab resulted in the identification of five unique peptides: HC-T_5_ GLVWVSAITSSGGSTYYSDTVK, HC-T_11_ LPDYWGQGTLVTVSSASTK, LC-T_3_ ASSSVSSSYL HWYQQKPGQAPR, LC-T_4_ LLIYSTSNLVAGIPDR and LC-T_6_ LEPEDFAVYYCQHHSGYHFTFGGGTK. All of them were detected in in-solution digests of Landogrozumab, and based on their detectability and location in the antibody, HC-T_5_ and LC-T_4_ were finally chosen as target peptides. These peptides were also successfully employed for the detection of the drug by Cheung et al. from equine plasma^38^. Especially HC-T_11_ and LC-T_6_ could also be valuable for analyte confirmation in case of an Adverse Analytical Finding (AAF). For the human antibody Stamulumab, the ProteinBlast search yielded a total of four peptides without matches in the UniProtKB/Swiss-Prot and ‘nr’ databases: HC-T_10_ DENWGFDPWGQGTLVTVSSASTK, LC-T_1_ SYELTQPPSVSVSPGQTASITCSGHALGDK, LC-T_2_ FVSWYQQKPGQ SPVLVIYDDTQRPSGIPER, and LC-T_3_ FSGSNSGNTATLTISGTQAMDEADYYCQAWDSSFVFGGGTK. As only peptides LC-T_1_ and LC-T_2_ were detected in in-solution digests of reference material, they were further evaluated as diagnostic peptides for the mass spectrometric detection of Stamulumab. In 2019, a specific mass spectrometric assay for the detection of the humanized anti-MSTN antibody Domagrozumab was described, which employs peptides HC-T_4_ GLEWVSTISSGGSYTSYPDSVK, HC-T_18_ THTCPPCPAPEAAGAPSVFLFPPKPK, and LC-T_3_ ASQDVSTAVAWYQQKPGK for identification purposes^29^. Peptides HC-T_4_ and LC-T_3_ were also recently used by two other groups for the specific detection of the antibody in human and equine blood samples^30,38^. Besides HC-T_4_ and HC-T_18_, only HC-T_10_ QDYAMNYWGQGTLVTVSSASTK and LC-T_7_ FSGSGSGTFFTLTISSLQPEDFATYYCQQHYSTPWTFGGGTK yielded no matches in both the UniProtKB/Swiss-Prot and ‘nr’ databases. All peptides except for LC-T_7_ were characterized by good detectability, and HC-T_4_ and HC-T_18_ were finally chosen for method development and validation. For confirmation purposes, HC-T_10_ and LC-T_3_ could also be evaluated. For the fully human antibody Apitegromab, the ProteinBlast search yielded only two tryptic peptides without any matches in the UniProtKB/Swiss-Prot and ‘nr’ databases: HC-T_13_ FLEWSHYYGMDVWGQGTTVTVSSASTK and LC-T_6_ LTVLGQPK. As their analytical response from in-solution digests of the therapeutic antibody was modest, LC-T_2_ VTISCSGSSSNIGSNTVHWYQQLPGTAPK and LC-T_3_ LLIYSDNQRPSGVPDR were selected as alternative targets for the proposed initial testing procedure (ITP). For both peptides, only the ‘nr’ database search yielded matches, which were however not detected in an exemplary blank serum extract, suggesting their specificity for the target protein. Yet, peptide HC-T_13_ might also be considered for confirmation purposes.

**Fig. 2:**
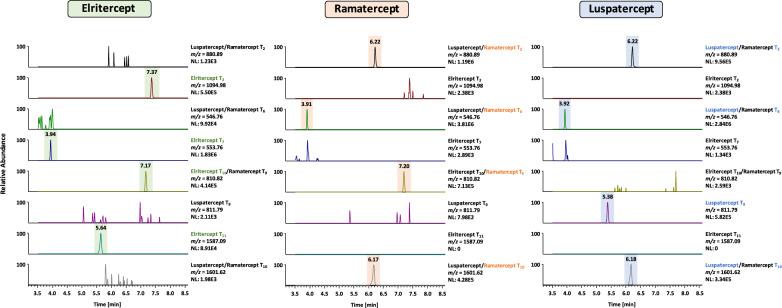
Extracted ion chromatograms acquired by tSIM used for the combined evaluation regarding the differentiation of Ramatercept, Luspatercept and Elritercept in a serum sample.

Sotatercept, Elritercept, Ramatercept and Luspatercept represent recombinant fusion proteins derived from the extracellular domains of the human ActRIIA/B and the Fc fragment of human IgG. Between both sequences, a short artificial linker is present. Diagnostic peptides spanning all three sections of the molecules would be ideal to provide evidence for the presence of the artificial fusion proteins in a sample as the respective amino acid sequences would be foreign to the human body, as indicated in an earlier study. ^19^ Unfortunately, the diagnostic fusion peptides obtained by in silico tryptic digestion of the analytes were either very long (Sotatercept, Luspatercept, Ramatercept) or contained a methionine residue prone to oxidation (Elritercept) and their presumed poor detectability was confirmed by analyzing in solution digests of the reference material. But the presence of a peptide derived from the extracellular receptor domains in the blood is highly indicative of the administration of the ActRII-Fc fusion proteins, as both ActRIIA and ActRIIB are large transmembrane proteins found on a variety of human cells but not freely circulating in the blood ^1^. For the ActRIIA-Fc fusion protein Sotatercept, a mass spectrometric detection method based on the diagnostic peptides T_2_ SETQECLFFNANWEK, located in the extracellular domain of human ActRIIA, and T_24_ ALPVPIEK, derived from the Fc fragment of human IgG1, was already published in 2016^19^. In peptide T_24_, a characteristic amino acid exchange (A → V) is present, which has not been reported to naturally occur in humans. Therefore, it can also be employed for the detection of Sotatercept in human serum or plasma. The ActRIIB-Fc fusion protein Ramatercept and its derivative Luspatercept differ only in an amino acid exchange (L → D) located in the receptor domain of the fusion protein. Consequently, only peptide T_9_ GCWLDDFNCYDR/GCWDDDFNCYDR can be used for an unambiguous differentiation and detection of both proteins^22^. While the ProteinBlast of the modified sequence demonstrated its uniqueness, the original tryptic peptide is also generated from the modified ActRIIA-Fc fusion protein Elritercept. Consequently, a combination of different diagnostic peptides has to be evaluated to distinguish between Luspatercept, Ramatercept and Elritercept. As shown in Fig. 2, peptides T_2_ SETQECLFYNANWELER, T_7_ LHCYATWR and T_11_ TDCVETEENPQVYFCCCEGNMCNEK in combination with T_10_ GCWLDDFNCYDR allow for an unambiguous identification of Elritercept while T_2_ ECIYYNANWELER, T_6_ LHCYASWR, T_9_ GCWLDDFNCYDR and T_10_ QECVATEENPQVYFCCCEGNFCNER are characteristic of Ramatercept. When Luspatercept is present in a sample, T_2_ ECIYYNANWELER, T_6_ LHCYASWR, T_9_ GCWDDDFNCYDR and T_10_ QECVATEENPQVYFCCCEGNFCNER can be detected.

### Method characterization

**Fig. 3:**
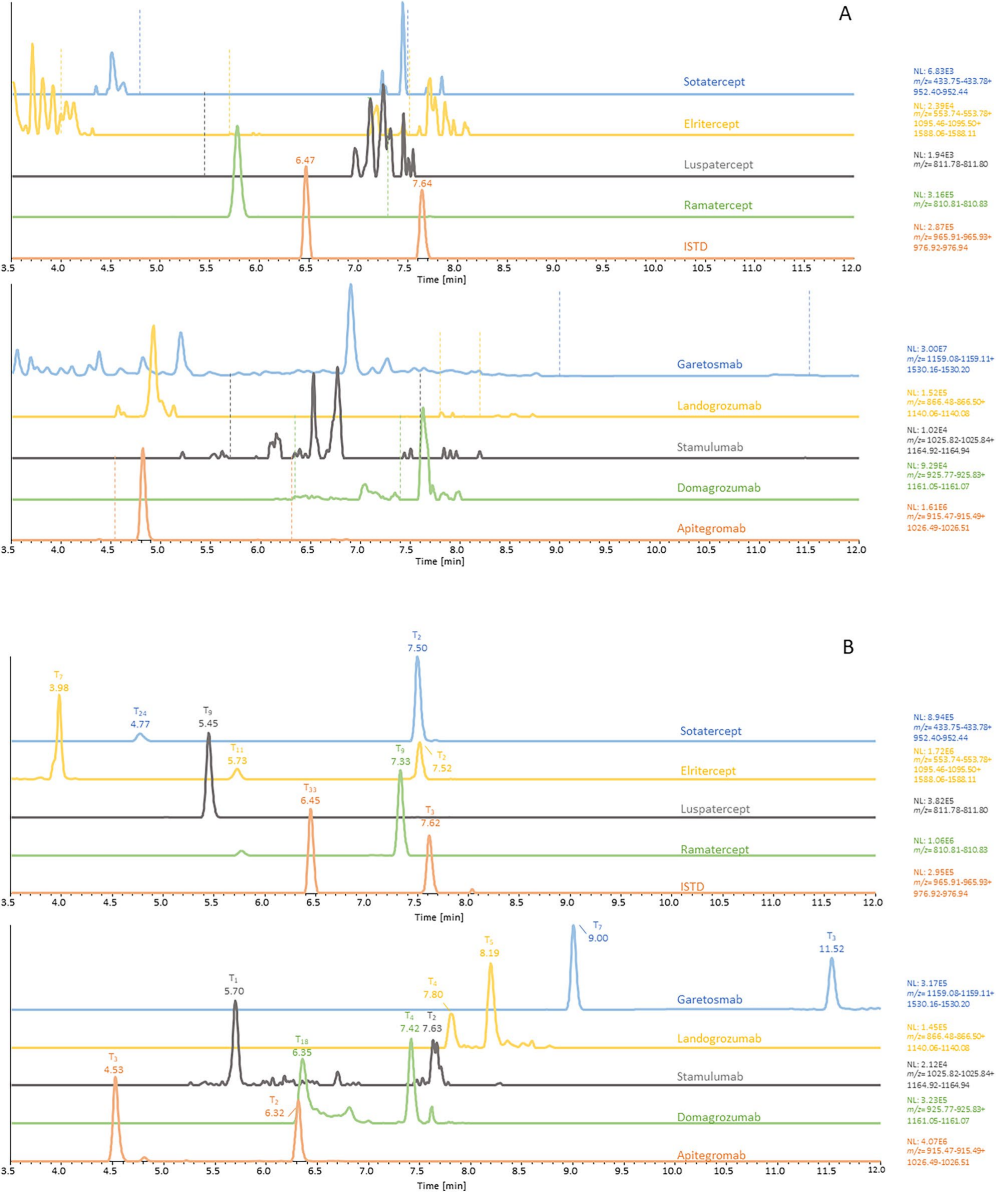
Representative extracted ion chromatograms acquired by tSIM of the target peptides of the nine IASPs and the ISTD of (**A**) one blank serum sample and (**B**) one serum sample spiked at a concentration of 100 ng/mL.

An extensive method validation, for the previously described diagnostic peptides, was successfully carried out, according to current WADA guidelines, proving the method applicability to doping control testing^31^. As shown in Table 2, the analytical method was characterised by a high selectivity for the IASPs Garetosmab, Sotatercept, Elritercept, Luspatercept, Ramatercept, Apitegromab, Landogrozumab, Stamulumab and Domagrozumab, with all samples showing a negative result, while the ISTD in all samples and the IASPs in a quality control sample were detected, demonstrating the validity of the analysis. Representative extracted ion chromatograms acquired by tSIM of the target peptides of the nine IASPs and ISTD of one blank sample are presented in Fig. 3A. For the human therapeutic antibodies Garetosmab and Apitegromab, signals were detectable in individual samples for one of the two diagnostic peptides (Garetosmab; LC T_7_ and Apitegromab; LC T_3_), which indicate a natural occurrence of the amino acid sequence in endogenous antibodies. However, due to the specificity of the second diagnostic peptide, this can be neglected in the context of the ITP. The evaluation of the spiked serum samples showed that all target analytes were detectable in the ten samples fortified with 100 ng/mL, proving the method’s reliability at this concentration. Representative extracted ion chromatograms acquired by tSIM of the target peptides of the nine IASPs at a concentration of 100 ng/mL are presented in Fig. 3B. For the calculation of the LODs, all peptides listed in Table 1 were evaluated and the LOD was defined as the lowest concentration at which all peptides for each analyte could be successfully detected (bold numbers in Table 2). The LOD was estimated as 10 ng/mL for Sotatercept, Luspatercept, Ramatercept and Domagrozumab, 25 ng/mL for Garetosmab, Elritercept, Landogrozumab and Stamulumab, and 50 ng/mL for Apitegromab. Method sensitivity can be further improved by increasing the sample volume and/or injection volume, if desirable. The sample carryover during the LC-HRMS/MS analysis was calculated at up to 10.4%, after the injection of a highly concentrated sample (500 ng/mL) followed by a blank sample. In the rare case that two suspicious samples for one analyte appear consecutively within the same analytical batch, particularly for analytes that exhibited higher carryover values during method validation, these samples’ reanalysis, with the inclusion of two blank samples between each suspicious sample, would help to mitigate any potential impact of carryover on result interpretation and ensure the reliability of the findings. The analysis of the magnetic beads washing fractions yielded no carryover, demonstrating that re-usage of the beads after thorough washing (e.g. three times with 300 µL of 3% acetic acid) is possible for concentrations up to 500 ng/mL. But as higher drug concentrations could result in significant carryover, it is recommended to re-use only the beads of negative samples for doping control routine applications. The method was found to be robust, as it enabled the detection of all target analytes also in plasma samples. The validation was performed by two different analysts on different days, enhancing the method robustness. Extract stability in the autosampler at 10 °C was demonstrated for concentrations equal to or higher than 10 ng/mL for at least 3 days. In addition, the assay was found to be linear from 10 to 500 ng/mL for Sotatercept, Elritercept, Luspatercept, Ramatercept, Apitegromab and Domagrozumab, and from 25 to 500 ng/mL for Garetosmab, Landogrozumab and Stamulumab. However, the degree of linearity was found to significantly differ between analytes as reflected by mean coefficients of correlation varying between 0.909 and 0.995. This difference can potentially be explained by saturation of the magnetic beads at higher drug concentrations. As all analytes were simultaneously spiked to the linearity samples and some of the protein drugs compete for the same TGF-β ligands (e.g. Landogrozumab, Domagrozumab & Stamulumab), a higher affinity of a drug for one of the ligands could impair the recovery of other analytes.

**Table 2:** Results of method validation.

Validation parameter:	n	Amount [ng]	Concentration(s) [ng/mL]	Garetosmab	Sotatercept	Elritercept	Luspatercept	Ramatercept	Apitegromab	Landogrozumab	Stamulumab	Domagrozumab
Selectivity	10	–	–	0/10	0/10	0/10	0/10	0/10	0/10	0/10	0/10	0/10
Reliability	10	20	100	10/10	10/10	10/10	10/10	10/10	10/10	10/10	10/10	10/10
LOD	6	2	10	3/6	**6/6**	5/6	**6/6**	**6/6**	4/6	4/6	3/6	**6/6**
	6	5	25	**6/6**	6/6	**6/6**	6/6	6/6	4/6	**6/6**	**6/6**	6/6
	6	10	50	6/6	6/6	6/6	6/6	6/6	**6/6**	6/6	6/6	6/6
	6	20	100	6/6	6/6	6/6	6/6	6/6	6/6	6/6	6/6	6/6
Robustness	10	20	100	10/10	10/10	10/10	10/10	10/10	10/10	10/10	10/10	10/10
Carryover (magnetic beads)	2	100	500	Yes*	Yes*	Yes*	Yes*	Yes*	Yes*	Yes*	Yes*	Yes*
Carryover (LC-HRMS/MS)	1	100	500	< 10%	< 5%	< 7%	< 5%	< 5%	< 1%	< 11%	–	< 5%
Linearity	8	2–100	10–500	25–500; R^2^ ≥ 0.985	10–500; R^2^ ≥ 0.954	10–500; R^2^ ≥ 0.909	10–500; R^2^ ≥ 0.995	10–500; R^2^ ≥ 0.995	10–500; R^2^ ≥ 0.989	25–500; R^2^ ≥ 0.971	25–500; R^2^ ≥ 0.929	10–500; R^2^ ≥ 0.993
Sample extract stability	8	2–100	10–500	≥ 3 d	≥ 3 d	≥ 3 d	≥ 3 d	≥ 3 d	≥ 3 d	≥ 3 d	≥ 3 d	≥ 3 d

Significant values for LOD estimation are in bold.

*At high drug concentrations > 500 ng/mL.

### Proof-of-concept: analysis of post-administration samples

**Fig. 4:**
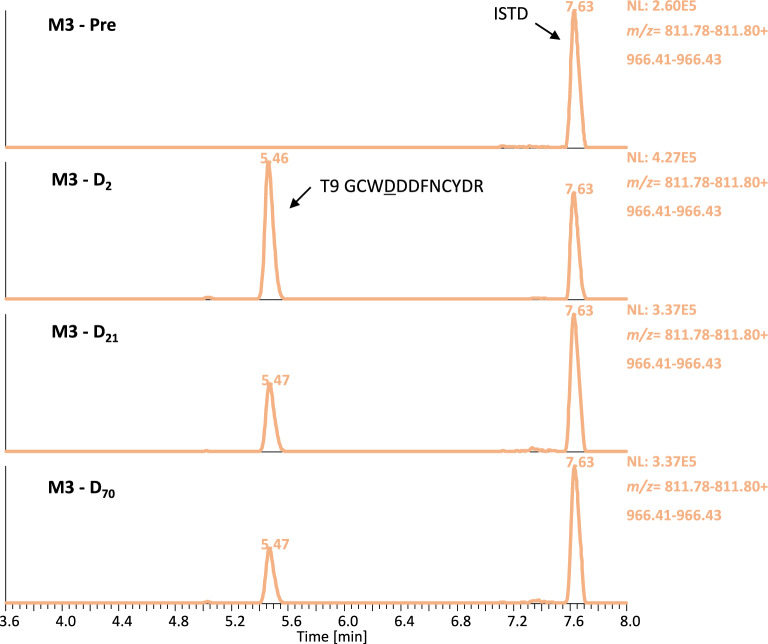
Extracted ion chromatograms acquired by tSIM of Luspatercept peptide T_9_ and the ISTD in pre- and post- administration serum samples of volunteer M3.

**Fig. 5:**
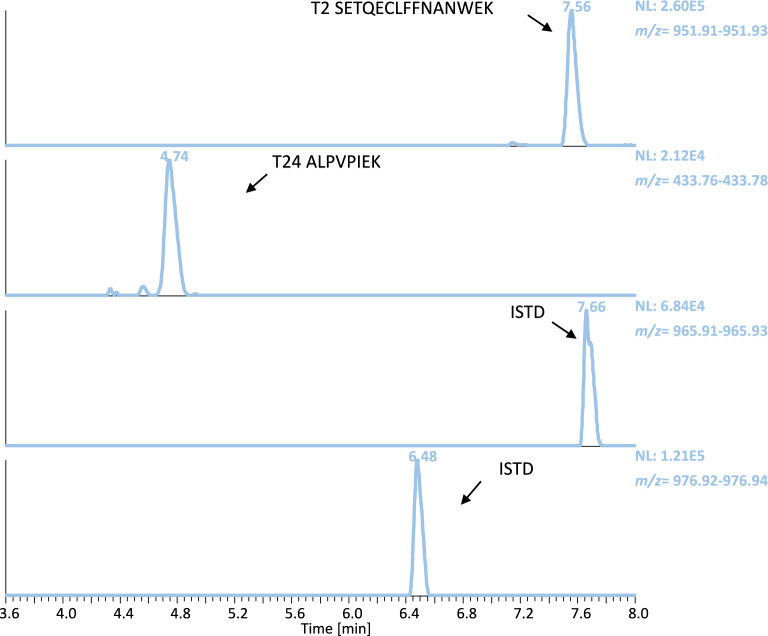
Extracted ion chromatograms acquired by tSIM of Sotatercept peptides T_2_ & T_24_ and the ISTD of pre- and 43 h post- administration serum samples.

The analysis of authentic samples from in vivo studies is desirable to establish proof-of-concept, ensuring that a prohibited substance or its biomarker can be detected using a certain analytical approach in doping control testing. Among the nine IASPs of interest, only two have already been approved by the U.S. Food and Drug Administration (FDA). Luspatercept was approved under the brand name Reblozyl^®^ for the treatment in certain conditions of anemia associated with beta-thalassemia (2019) and anemia associated with myelodysplastic syndromes (2020)^39,40^. Sotatercept has also received regulatory approval under the brand name Winrevair^®^ for the treatment of pulmonary arterial hypertension (2024)^41^. Moreover, Luspatercept and Sotatercept are included in the WADA Prohibited List and are of particular interest for the WADA accredited laboratories^8^. As a result, analysis of post-administration samples for these two therapeutic drugs was considered essential. Using the herein reported approach, the diagnostic peptide of Luspatercept (T_9_ GCWDDDFNCYDR) was detectable in the serum samples up to 70 days after the first dose and 49 days after the second in the four volunteers (Table 3A & Fig. 4). The serum maximum concentrations were estimated from 1235.5 to 1664.8 ng/mL (Supporting Information Fig. S23), suggesting that there might be inter-individual differences regarding the drug’s bioavailability, and their pharmacokinetic profile was in good agreement with the results obtained by Marchand et al.^27^. In addition, both diagnostic peptides of Sotatercept (T_2_ SETQECLFFNANWEK and T_24_ ALPVPIEK) were unambiguously detected in a serum sample collected 43 h following injection of the fusion protein (Fig. 5). The corresponding serum concentration was estimated at 338.4 ng/mL. The successful detection of Luspatercept and Sotatercept clearly demonstrates the applicability of the detection method described herein to authentic post-administration serum samples, enhancing its ability to be implemented in routine doping control analysis.

**Table 3:**
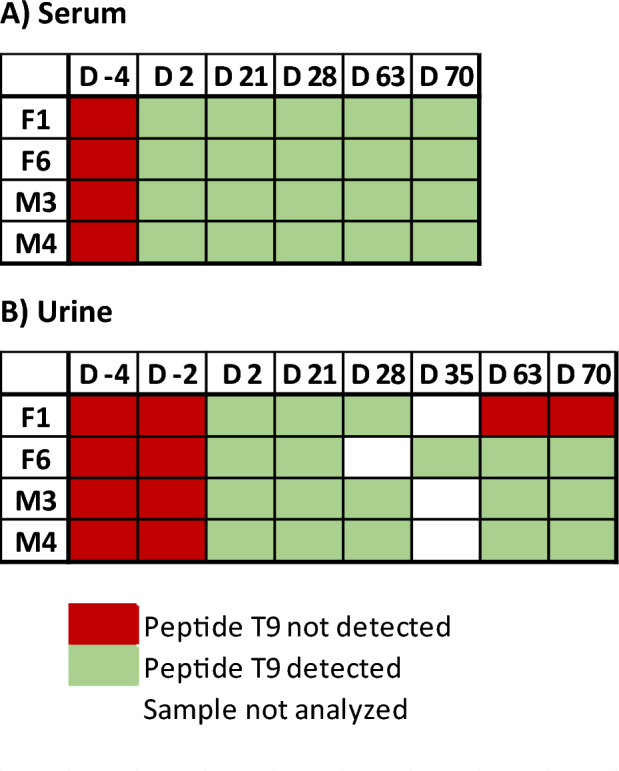
Detectability of the Luspatercept target peptide T_9_ in the pre- and post-administration serum (A) and urine (B) samples.

### Outlook: urine as alternative biological matrix?

During the Luspatercept administration study conducted by Marchand et al. also urine specimens were collected to investigate the urinary elimination of the fusion protein^27^. According to the European Medicines Agency (EMA), the drug is not expected to be excreted into urine as is molecular mass is above the size exclusion threshold of the glomerular filtration barrier^42^. Nevertheless, Marchand et al. could clearly detect the drug in all post-administration urine samples for at least 70 days after the first dose and 49 days after the second by using different electrophoretic techniques and immunoblotting. The detected urinary concentrations were estimated to be by a factor of 100 to 1000 lower than in the corresponding serum specimens^27^.

**Fig. 6:**
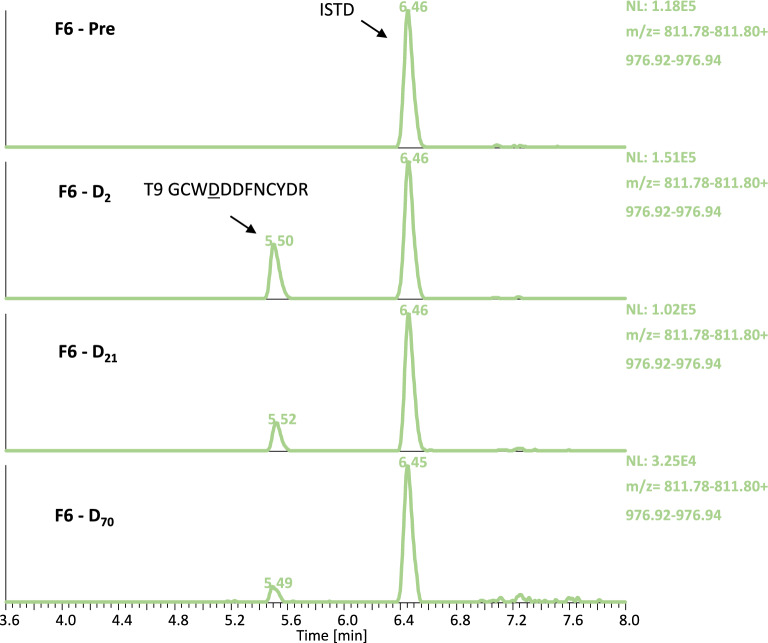
Extracted ion chromatograms acquired by tSIM of Luspatercept peptide T_9_ and the ISTD in pre- and post- administration urine samples of volunteer F6.

Within this research project, selected pre- and post-administration urine samples were extracted with a modified protocol of the method described above. To concentrate the urine specimens prior to (immuno-)affinity purification, tryptic digestion and LC-HRMS/MS analysis, the ammonium sulfate precipitation step was replaced with the ultrafiltration using Amicon^®^ centrifugal filter units with a cut-off of 10 kDa. As shown in Table 3B and Fig. 6, Luspatercept diagnostic peptide T_9_ (GCWDDDFNCYDR) was detected in three subjects (M3, M4 and F6) for at least 70 days after the first dose and 49 days after the second, and in one volunteer (F1) for a minimum of 28 days. The corresponding maximum concentrations were estimated to range from 0.92 to 4.14 ng/mL. Consequently, also urine could be employed as biological matrix for at least one of the target analytes.

## Conclusion

Within this study, a multiplexed assay for the combined detection of nine different IASPs (Garetosmab, Sotatercept, Elritercept, Luspatercept, Ramatercept, Apitegromab, Landogrozumab, Stamulumab and Domagrozumab) in doping control serum and plasma samples using LC-HRMS/MS was developed and successfully validated according to WADA guidelines. These therapeutic drugs or candidate drugs are usually recommended to be administered at high doses (µg/kg body weight). As a result, the LOD of 10 to 50 ng/mL should enable long detection windows, likely spanning over several weeks. Authentic Luspatercept and Sotatercept post-administration serum samples were successfully analyzed as proof-of-concept. The method was modified to allow for the use of urine instead of serum, and Luspatercept post-administration urine samples were also analyzed. The present method could be employed in doping control routine analysis as an initial testing procedure enabling both the clear identification of the target analytes at the amino acid level and a retrospective evaluation of the samples for the presence of novel IASPs binding to one of the TGF-β cytokines or the anti-ActRIIB antibody used during (immuno-)affinity purification. It would be beneficial to develop confirmation methods. These methods would likely require dedicated modifications to both the sample preparation procedure and the LC–MS/MS method, thereby distinguishing them from the presented initial testing procedure. For instance, magnetic beads could be conjugated with a single cytokine or antibody, different proteases may be used for digestion and the mass spectrometric analysis could be tailored to selectively target the tryptic peptides of the specific analyte under investigation for confirmation.

## Supplementary Information

Supplementary Information.

## Data Availability

All MS data generated during the current study are available from the corresponding authors on reasonable request.
