# Supplementary material for: Combined detection of inhibitors of the activin receptor signaling pathways (IASPs) by means of LC-HRMS/MS for human doping control
Source: Sci Rep. 2025 Jun 6;15:19887. doi: 10.1038/s41598-025-03562-y (PMC12144231; doi:10.1038/s41598-025-03562-y)
Supplement: Supplementary file 1 — Supplementary Information. [file 41598_2025_3562_MOESM1_ESM.docx]

Supporting Information

**Combined detection of inhibitors of the activin receptor signaling pathways (IASPs) by means of LC-HRMS/MS for human doping control**

Panagiotis Sakellariou^a*+^, Katja Walpurgis^a*+^, Andreas Thomas^a^, Alexandre Marchand^b^, Geoff Miller^c^, Frank Dellanna^d^, Mario Thevis^a, e^

^a^ Institute of Biochemistry/Center for Preventive Doping Research, German Sport University Cologne, Cologne, Germany

^b^ Laboratoire Antidopage Français (LADF), Université Paris-Saclay, Orsay, France

^c^ Sports Medicine Research and Testing Laboratory (SMRTL), South Jordan, Utah, USA

^d^ MVZ DaVita Rhein-Ruhr GmbH, Bismarckstraße 101, 40210 Düsseldorf, Germany

^e^ European Monitoring Center for Emerging Doping Agents (EuMoCEDA), Cologne/Bonn, Germany

^+^ Equal contribution

**Overview**

**Figure S1** Product ion mass spectrum of tryptic peptide T_3_ ([M+2H]^2+^: *m/z* = 1158.60) diagnostic for the heavy chain of the therapeutic antibody Garetosmab. The spectrum was acquired on an Orbitrap Exploris^TM^ 480 coupled to a Vanquish^TM^ UHPLC.

**Figure S2** Product ion mass spectrum of tryptic peptide T_7_ ([M+2H]^2+^: *m/z* = 1529.68) diagnostic for the light chain of the therapeutic antibody Garetosmab. The spectrum was acquired on an Orbitrap Exploris^TM^ 480 coupled to a Vanquish^TM^ UHPLC.

**Figure S3** Product ion mass spectrum of tryptic peptide T_2_ ([M+2H]^2+^: *m/z* = 951.92) diagnostic for the ActRIIA-Fc fusion protein Sotatercept. The spectrum was acquired on an Orbitrap Exploris^TM^ 480 coupled to a Vanquish^TM^ UHPLC.

**Figure S4** Product ion mass spectrum of tryptic peptide T_24_ ([M+2H]^2+^: *m/z* = 433.77) diagnostic for the ActRIIA-Fc fusion protein Sotatercept. The spectrum was acquired on an Orbitrap Exploris^TM^ 480 coupled to a Vanquish^TM^ UHPLC.

**Figure S5** Product ion mass spectrum of tryptic peptide T_2_ ([M+2H]^2+^: *m/z* = 1094.98) diagnostic for the modified ActRIIA-Fc fusion protein Elritercept. The spectrum was acquired on an Orbitrap Exploris^TM^ 480 coupled to a Vanquish^TM^ UHPLC.

**Figure S6** Product ion mass spectrum of tryptic peptide T_7_ ([M+2H]^2+^: *m/z* = 553.76) diagnostic for the modified ActRIIA-Fc fusion protein Elritercept. The spectrum was acquired on an Orbitrap Exploris^TM^ 480 coupled to a Vanquish^TM^ UHPLC.

**Figure S7** Product ion mass spectrum of tryptic peptide T_10_ ([M+2H]^2+^: *m/z* = 810.81) of the modified ActRIIA-Fc fusion protein Elritercept and T_9_ of the ActRIIB-Fc fusion protein Ramatercept. The spectrum was acquired on an Orbitrap Exploris^TM^ 480 coupled to a Vanquish^TM^ UHPLC.

**Figure S8** Product ion mass spectrum of tryptic peptide T_11_ ([M+2H]^2+^: *m/z* = 1587.09) diagnostic for the modified ActRIIA-Fc fusion protein Elritercept. The spectrum was acquired on an Orbitrap Exploris^TM^ 480 coupled to a Vanquish^TM^ UHPLC.

**Figure S9** Product ion mass spectrum of tryptic peptide T_2_ ([M+2H]^2+^: *m/z* = 880.39) of the ActRIIB-Fc fusion protein Ramatercept and the modified ActRIIB-Fc fusion protein Luspatercept. The spectrum was acquired on an Orbitrap Exploris^TM^ 480 coupled to a Vanquish^TM^ UHPLC.

**Figure S10** Product ion mass spectrum of tryptic peptide T_6_ ([M+2H]^2+^: *m/z* = 546.76) of the ActRIIB-Fc fusion protein Ramatercept and the modified ActRIIB-Fc fusion protein Luspatercept. The spectrum was acquired on an Orbitrap Exploris^TM^ 480 coupled to a Vanquish^TM^ UHPLC.

**Figure S11** Product ion mass spectrum of tryptic peptide T_9_ ([M+2H]^2+^: *m/z* = 811.79) diagnostic for the modified ActRIIB-Fc fusion protein Luspatercept. The spectrum was acquired on an Orbitrap Exploris^TM^ 480 coupled to a Vanquish^TM^ UHPLC.

**Figure S12** Product ion mass spectrum of tryptic peptide T_10_ ([M+2H]^2+^: *m/z* = 1600.62) of the ActRIIB-Fc fusion protein Ramatercept and the modified ActRIIB-Fc fusion protein Luspatercept. The spectrum was acquired on an Orbitrap Exploris^TM^ 480 coupled to a Vanquish^TM^ UHPLC.

**Figure S13** Product ion mass spectrum of tryptic peptide T_2_ ([M+3H]^3+^: *m/z* = 1026.17) diagnostic for the light chain of the therapeutic antibody Apitegromab. The spectrum was acquired on an Orbitrap Exploris^TM^ 480 coupled to a Vanquish^TM^ UHPLC.

**Figure S14** Product ion mass spectrum of tryptic peptide T_3_ ([M+2H]^2+^: *m/z* = 915.48) diagnostic for the light chain of the therapeutic antibody Apitegromab. The spectrum was acquired on an Orbitrap Exploris^TM^ 480 coupled to a Vanquish^TM^ UHPLC.

**Figure S15** Product ion mass spectrum of tryptic peptide T_5_ ([M+2H]^2+^: *m/z* = 1139.57) diagnostic for the heavy chain of the therapeutic antibody Landogrozumab. The spectrum was acquired on an Orbitrap Exploris^TM^ 480 coupled to a Vanquish^TM^ UHPLC.

**Figure S16** Product ion mass spectrum of tryptic peptide T_4_ ([M+2H]^2+^: *m/z* = 866.49) diagnostic for the light chain of the therapeutic antibody Landogrozumab. The spectrum was acquired on an Orbitrap Exploris^TM^ 480 coupled to a Vanquish^TM^ UHPLC.

**Figure S17** Product ion mass spectrum of tryptic peptide T_1_ ([M+3H]^3+^: *m/z* = 1025.50) diagnostic for the light chain of the therapeutic antibody Stamulumab. The spectrum was acquired on an Orbitrap Exploris^TM^ 480 coupled to a Vanquish^TM^ UHPLC.

**Figure S18** Product ion mass spectrum of tryptic peptide T_2_ ([M+3H]^3+^: *m/z* = 1164.26) diagnostic for the light chain of the therapeutic antibody Stamulumab. The spectrum was acquired on an Orbitrap Exploris^TM^ 480 coupled to a Vanquish^TM^ UHPLC.

**Figure S19** Product ion mass spectrum of tryptic peptide T_4_ ([M+2H]^2+^: *m/z* = 1160.55) diagnostic for the heavy chain of the therapeutic antibody Domagrozumab. The spectrum was acquired on an Orbitrap Exploris^TM^ 480 coupled to a Vanquish^TM^ UHPLC.

**Figure S20** Product ion mass spectrum of tryptic peptide T_18_ ([M+3H]^3+^: *m/z* = 925.46) diagnostic for the heavy chain of the therapeutic antibody Domagrozumab. The spectrum was acquired on an Orbitrap Exploris^TM^ 480 coupled to a Vanquish^TM^ UHPLC.

**Figure S21** Product ion mass spectrum of tryptic peptide T_3_ ([M+2H]^2+^: *m/z* = 965.92) of the ISTD (mActRIIA-mFc). The spectrum was acquired on an Orbitrap Exploris^TM^ 480 coupled to a Vanquish^TM^ UHPLC.

**Figure S22** Product ion mass spectrum of tryptic peptide T_33_ ([M+2H]^2+^: *m/z* = 976.93) of the ISTD (mActRIIA-mFc). The spectrum was acquired on an Orbitrap Exploris^TM^ 480 coupled to a Vanquish^TM^ UHPLC.

**Figure S23** Luspatercept Administration Study: Estimated drug concentrations

**Figure S1 -** Product ion mass spectrum of tryptic peptide T_3_ ([M+2H]^2+^: *m/z* = 1158.60) diagnostic for the heavy chain of the therapeutic antibody Garetosmab. The spectrum was acquired on an Orbitrap Exploris^TM^ 480 coupled to a Vanquish^TM^ UHPLC.

**Figure S2 -** Product ion mass spectrum of tryptic peptide T_7_ ([M+2H]^2+^: *m/z* = 1529.68) diagnostic for the light chain of the therapeutic antibody Garetosmab. The spectrum was acquired on an Orbitrap Exploris^TM^ 480 coupled to a Vanquish^TM^ UHPLC.

**Figure S3 -** Product ion mass spectrum of tryptic peptide T_2_ ([M+2H]^2+^: *m/z* = 951.92) diagnostic for the ActRIIA-Fc fusion protein Sotatercept. The spectrum was acquired on an Orbitrap Exploris^TM^ 480 coupled to a Vanquish^TM^ UHPLC.

**Figure S4 -** Product ion mass spectrum of tryptic peptide T_24_ ([M+2H]^2+^: *m/z* = 433.77) diagnostic for the ActRIIA-Fc fusion protein Sotatercept. The spectrum was acquired on an Orbitrap Exploris^TM^ 480 coupled to a Vanquish^TM^ UHPLC.

**Figure S5 -** Product ion mass spectrum of tryptic peptide T_2_ ([M+2H]^2+^: *m/z* = 1094.98) diagnostic for the modified ActRIIA-Fc fusion protein Elritercept. The spectrum was acquired on an Orbitrap Exploris^TM^ 480 coupled to a Vanquish^TM^ UHPLC.

**Figure S6 -** Product ion mass spectrum of tryptic peptide T_7_ ([M+2H]^2+^: *m/z* = 553.76) diagnostic for the modified ActRIIA-Fc fusion protein Elritercept. The spectrum was acquired on an Orbitrap Exploris^TM^ 480 coupled to a Vanquish^TM^ UHPLC.

**Figure S7 -** Product ion mass spectrum of tryptic peptide T_10_ ([M+2H]^2+^: *m/z* = 810.81) of the modified ActRIIA-Fc fusion protein Elritercept and T_9_ of the ActRIIB-Fc fusion protein Ramatercept. The spectrum was acquired on an Orbitrap Exploris^TM^ 480 coupled to a Vanquish^TM^ UHPLC.

**Figure S8 -** Product ion mass spectrum of tryptic peptide T_11_ ([M+2H]^2+^: *m/z* = 1587.09) diagnostic for the modified ActRIIA-Fc fusion protein Elritercept. The spectrum was acquired on an Orbitrap Exploris^TM^ 480 coupled to a Vanquish^TM^ UHPLC.

**Figure S9 -** Product ion mass spectrum of tryptic peptide T_2_ ([M+2H]^2+^: *m/z* = 880.39) of the ActRIIB-Fc fusion protein Ramatercept and the modified ActRIIB-Fc fusion protein Luspatercept. The spectrum was acquired on an Orbitrap Exploris^TM^ 480 coupled to a Vanquish^TM^ UHPLC.

**Figure S10 -** Product ion mass spectrum of tryptic peptide T_6_ ([M+2H]^2+^: *m/z* = 546.76) of the ActRIIB-Fc fusion protein Ramatercept and the modified ActRIIB-Fc fusion protein Luspatercept. The spectrum was acquired on an Orbitrap Exploris^TM^ 480 coupled to a Vanquish^TM^ UHPLC.

**Figure S11 -** Product ion mass spectrum of tryptic peptide T_9_ ([M+2H]^2+^: *m/z* = 811.79) diagnostic for the modified ActRIIB-Fc fusion protein Luspatercept. The spectrum was acquired on an Orbitrap Exploris^TM^ 480 coupled to a Vanquish^TM^ UHPLC.

**Figure S12 -** Product ion mass spectrum of tryptic peptide T_10_ ([M+2H]^2+^: *m/z* = 1600.62) of the ActRIIB-Fc fusion protein Ramatercept and the modified ActRIIB-Fc fusion protein Luspatercept. The spectrum was acquired on an Orbitrap Exploris^TM^ 480 coupled to a Vanquish^TM^ UHPLC.

**Figure S13 -** Product ion mass spectrum of tryptic peptide T_2_ ([M+3H]^3+^: *m/z* = 1026.17) diagnostic for the light chain of the therapeutic antibody Apitegromab. The spectrum was acquired on an Orbitrap Exploris^TM^ 480 coupled to a Vanquish^TM^ UHPLC.

**Figure S14 -** Product ion mass spectrum of tryptic peptide T_3_ ([M+2H]^2+^: *m/z* = 915.48) diagnostic for the light chain of the therapeutic antibody Apitegromab. The spectrum was acquired on an Orbitrap Exploris^TM^ 480 coupled to a Vanquish^TM^ UHPLC.

**Figure S15 -** Product ion mass spectrum of tryptic peptide T_5_ ([M+2H]^2+^: *m/z* = 1139.57) diagnostic for the heavy chain of the therapeutic antibody Landogrozumab. The spectrum was acquired on an Orbitrap Exploris^TM^ 480 coupled to a Vanquish^TM^ UHPLC.

**Figure S16 -** Product ion mass spectrum of tryptic peptide T_4_ ([M+2H]^2+^: *m/z* = 866.49) diagnostic for the light chain of the therapeutic antibody Landogrozumab. The spectrum was acquired on an Orbitrap Exploris^TM^ 480 coupled to a Vanquish^TM^ UHPLC.

**Figure S17 -** Product ion mass spectrum of tryptic peptide T_1_ ([M+3H]^3+^: *m/z* = 1025.50) diagnostic for the light chain of the therapeutic antibody Stamulumab. The spectrum was acquired on an Orbitrap Exploris^TM^ 480 coupled to a Vanquish^TM^ UHPLC.

**Figure S18 -** Product ion mass spectrum of tryptic peptide T_2_ ([M+3H]^3+^: *m/z* = 1164.26) diagnostic for the light chain of the therapeutic antibody Stamulumab. The spectrum was acquired on an Orbitrap Exploris^TM^ 480 coupled to a Vanquish^TM^ UHPLC.

**Figure S19 -** Product ion mass spectrum of tryptic peptide T_4_ ([M+2H]^2+^: *m/z* = 1160.55) diagnostic for the heavy chain of the therapeutic antibody Domagrozumab. The spectrum was acquired on an Orbitrap Exploris^TM^ 480 coupled to a Vanquish^TM^ UHPLC.

**Figure S20 -** Product ion mass spectrum of tryptic peptide T_18_ ([M+3H]^3+^: *m/z* = 925.46) diagnostic for the heavy chain of the therapeutic antibody Domagrozumab. The spectrum was acquired on an Orbitrap Exploris^TM^ 480 coupled to a Vanquish^TM^ UHPLC.

**Figure S21 -** Product ion mass spectrum of tryptic peptide T_3_ ([M+2H]^2+^: *m/z* = 965.92) of the ISTD (mActRIIA-mFc). The spectrum was acquired on an Orbitrap Exploris^TM^ 480 coupled to a Vanquish^TM^ UHPLC.

**Figure S22 -** Product ion mass spectrum of tryptic peptide T_33_ ([M+2H]^2+^: *m/z* = 976.93) of the ISTD (mActRIIA-mFc). The spectrum was acquired on an Orbitrap Exploris^TM^ 480 coupled to a Vanquish^TM^ UHPLC.

**Figure S23 -** Luspatercept Administration Study: Estimated drug concentrations.
